# Effectiveness of zinc supplementation on diarrhea and average daily gain in pre-weaned dairy calves: A double-blind, block-randomized, placebo-controlled clinical trial

**DOI:** 10.1371/journal.pone.0219321

**Published:** 2019-07-10

**Authors:** Hillary R. Feldmann, Deniece R. Williams, John D. Champagne, Terry W. Lehenbauer, Sharif S. Aly

**Affiliations:** 1 Veterinary Medicine Teaching and Research Center, University of California, Davis School of Veterinary Medicine, Tulare, California, United States of America; 2 Department of Population Health and Reproduction, University of California, Davis School of Veterinary Medicine, Davis, California, United States of America; National Veterinary School of Toulouse, FRANCE

## Abstract

The objective of this clinical trial was to evaluate the effectiveness of zinc supplementation on diarrhea and average daily weight gain (ADG) in pre-weaned dairy calves. A total of 1,482 healthy Holstein heifer and bull calves from a large California dairy were enrolled at 24 to 48 hours of age until hutch exit at approximately 90 days of age. Calves were block-randomized by time to one of three treatments: 1) placebo, 2) zinc methionine (ZM), or 3) zinc sulfate (ZS) administered in milk once daily for 14 days. Serum total protein at enrollment and body weight at birth, treatment end, and hutch exit were measured. Fecal consistency was assessed daily for 28 days post-enrollment. For a random sample of 127 calves, serum zinc concentrations before and after treatment and a fecal antigen ELISA at diarrhea start and resolution for *Escherichia coli* K99, rotavirus, coronavirus, and *Cryptosporidium parvum* were performed. Linear regression showed that ZM-treated bull calves had 22 g increased ADG compared to placebo-treated bulls (*P* = 0.042). ZM-treated heifers had 9 g decreased ADG compared to placebo-treated heifers (*P* = 0.037), after adjusting for average birth weight. Sex-stratified models showed that high birth weight heifers treated with ZM gained more than placebo-treated heifers of the same birth weight, which suggests a dose-response effect rather than a true sex-specific effect of ZM on ADG. Cox regression showed that ZM and ZS-treated calves had a 14.7% (*P* = 0.015) and 13.9% (*P* = 0.022) reduced hazard of diarrhea, respectively, compared to placebo-treated calves. Calves supplemented for at least the first five days of diarrhea with ZM and ZS had a 21.4% (*P* = 0.027) and 13.0% (*P* = 0.040) increased hazard of cure from diarrhea, respectively, compared to placebo-treated calves. Logistic regression showed that the odds of microbiological cure at diarrhea resolution for rotavirus, *C*. *parvum*, or any single fecal pathogen was not different between treatment groups. Zinc supplementation delayed diarrhea and expedited diarrhea recovery in pre-weaned calves. Additionally, zinc improved weight gain differentially in bulls compared to heifers, indicating a research need for sex-specific dosing.

## Introduction

Diarrhea is the leading cause of morbidity and mortality and the most common reason for antimicrobial drug treatments in pre-weaned dairy heifers [1, 2]. A USDA survey of pre-weaned dairy heifers reported that 24% experienced diarrhea and 18% received antimicrobial treatment for it [1]. Diarrhea is also a leading cause of morbidity and the second foremost cause of mortality in children with over 1 billion cases and a half a million deaths annually [3, 4]. Zinc supplementation in children decreases the incidence, duration, and severity of diarrhea, increases recovery rates, decreases the use of antibiotics and antidiarrheal medications, and reduces mortality [5–10]. In a clinical trial that established a non-toxic zinc dose and investigated its therapeutic use for diarrhea in neonatal dairy calves, zinc-treated calves had numerically quicker clinical recovery, increased weight gain, and higher odds of fecal clearance of *Cryptosporidium parvum* between diarrhea onset and recovery compared with placebo-treated calves [11]. As a result, zinc supplementation may be beneficial for prevention of diarrhea in dairy calves and, thus, minimize antimicrobial use. However, studies investigating zinc’s potential effectiveness are lacking.

In children, both organic (zinc acetate, zinc gluconate, and zinc methionine) and inorganic (zinc sulfate and zinc oxide) zinc formulations are beneficial in the prevention and treatment of childhood diarrhea [12–15]. However, differing bioavailability was observed in several animal studies [16–19]. In addition, the underlying mechanism of action of oral zinc is unknown [6]. Hence, contrasting the effect, if any, of organic compared to inorganic zinc formulations in pre-weaned calves may help identify differences in mode of action.

The objective of this clinical trial was to compare average daily weight gain (ADG) and the incidence and duration of diarrhea in pre-weaned dairy calves randomly assigned to receive either organic zinc methionine (ZM), inorganic zinc sulfate (ZS), or a placebo in milk once daily for 14 days. By elucidating the potential role of zinc supplementation in prevention of diarrhea in pre-weaned dairy calves, calf morbidity, mortality, and antimicrobial usage may be mitigated.

## Materials and methods

### Study design and population

A double-blind, block randomized, placebo-controlled clinical trial was conducted between December 14, 2015 and June 15, 2016 on a large dairy in California’s San Joaquin Valley. The dairy was selected based on the owner and calf manager’s willingness to participate in the study. The dairy herd was composed of 5,500 lactating cows, predominantly Holsteins, and housed approximately 1,600 pre-weaned calves. Approximately 75% of the calves were born on the participating dairy and 25% were born on an affiliated dairy located approximately 10 miles away. Calves enrolled in the trial included healthy Holstein heifer or bull calves 24 to 48 hours of age. Calves were determined to be healthy via visual examination by a veterinarian (HF) or a trained researcher. Calves were excluded if they had obvious morbidities or congenital defects, were non-Holstein, born on the affiliated dairy, younger than 24 hours of age or older than 48 hours of age at the time of enrollment. Calves from the affiliated dairy were excluded due to differences in physical location and management practices of pre-partum cows. All procedures were approved by the University of California Davis Institutional Animal Care and Use Committee (protocol number 18067 *Approved*: *March 6*, *2014*).

### Sample size estimation

Results of a previous zinc clinical trial [11] indicated that a sample size of 213 diarrheic calves per group (α = 0.05, β = 0.10, power 90%) would be needed to show a difference in ADG of 107 g between treatment groups (Stata, College Station, TX). After allowing for 15% attrition and assuming 50% incidence of diarrhea based on study authors expert opinion, and a difference in ADG of 107g [11], a sample size of 500 calves per treatment group (*n* = 1500 total) was deemed required.

### Pre-weaned calf management

Newborn calves were removed from the dam within an hour of birth and placed in a straw-bedded, group calf pen where their navels were dipped in an iodine-based solution. Each calf received 4 liters of colostrum within 1 hour of birth and a second colostrum feeding (2 liters) 6–10 hours after birth. Colostrum was refrigerated for < 48 hours and heated in a hot water bath prior to feeding using an esophageal tube feeder. Within 18 hours of birth, pre-weaned calves were transported to individual metal hutches initially bedded with almond shells. Straw hay was later added to wet and muddy hutches throughout the pre-weaning period.

For the first 14 days of life, pre-weaned calves were bottle-fed 1.9 liters of milk twice daily and 1.9 liters of a commercial oral electrolyte solution (Calva Lyte; Calva Products, Inc., Acampo, CA) once daily between milk feedings. Milk consisted of a combination of pasteurized waste milk, rehydrated commercial milk replacer powder (Strauss Feeds LLC, Watertown, WI), tetracycline and neomycin powder, and additional supplements (S1 Table). The proportion of pasteurized waste milk to milk replacer varied with each feeding, as the volume of waste milk varied with changes in the number and production of cows contributing to the waste milk tank. After 14 days of age, calves were bottle-fed 2.8 liters of milk twice daily. Calves with clinical diarrhea received 1.9 liters of a commercial oral electrolyte solution (NuLife; Genex Cooperative, Inc., Shawano, WI) once daily between milk feedings. A list of ingredients that make up the two oral electrolyte solutions can be found in S2 Table. All pre-weaned calves had free choice access to water and a calf starter grain mix. Calves were gradually weaned over a 10-day period, starting at approximately 60 days of age after which calves received a grower grain mix until 90 days of age when they were moved to group pens.

Each calf received 1 mL of a selenium supplement (MU-SE; Merck Animal Health, Boxmeer, Netherlands) intramuscularly within 24 hours of age and an intranasal vaccine (Inforce 3, Zoetis, Inc., Florham Park, NJ) within 48 hours of age and again near the time of weaning. Approximately 8.3% (n = 126) of all enrolled calves were also vaccinated using an autogenous *Moraxella bovis*/*bovoculi* bacterin vaccine (Newport Laboratories, Inc., Worthington, MN) for the prevention of pinkeye at 5 and 7 weeks of age. All pre-weaned calves were evaluated daily by dairy personnel for calfhood diseases and treated according to standard on-farm treatment protocols. With regard to diarrhea therapy, calves less than two weeks of age with clinical diarrhea received an oral mixture of 118.5 mL (2.08 g) bismuth subsalicylate (Bismusal Suspension, Durvet, Inc., Blue Springs, MO) and 31.5 mL (1575 mg) spectinomycin (SpectoGard, Bimeda, Inc., Le Sueur, MN) once daily for two days. Calves older than two weeks of age with clinical diarrhea received oral sulfamethoxazole (1600 mg)/trimethoprim (320 mg) (Amneal Pharmaceuticls of NY, Hauppauge, NY) once daily for 2 to 3 days. Repeated treatment was at the discretion of the calf manager.

### Zinc treatment

Prior to the trial, total daily dietary zinc consumed by calves during the first 14 days of life was estimated using heavy metal analysis of water (n = 2), calf milk (n = 2), two types of oral electrolyte solutions (n = 2), calf starter grain (n = 2), and an estimated intake of starter grain by calves less than 14 days of age [20, 21]. Analysis was performed at the California Animal Health and Food Safety System’s (CAHFS) Toxicology Laboratory (Davis, CA) by ICP-MS (inductively coupled plasma mass spectrometry). Financial limitations restricted the ability to test more than two samples of each dietary component. Due to variation of zinc content in duplicate samples, the maximum concentration was used to estimate the daily zinc intake during the first 14 days of life (S3 Table).

In blocks of 36, enrolled calves were randomized using a random number generator (Microsoft Corporation, Redmond, WA) to one of three treatment groups: 1) placebo, 2) ZM, or 3) ZS to be administered in the morning milk feeding once daily for 14 days, starting on the day after enrollment. During the 14-day zinc treatment period, study calves that did not drink the entire milk bottle were tube-fed the remaining milk by trained technicians using an esophageal feeder disinfected between uses. The ZM treatment group received 0.45 g zinc methionine complex (equivalent to 80 mg of elemental zinc) as the product Zinpro180 (Zinpro Corporation, Eden Prairie, MN) combined with 0.44 g milk replacer powder. The ZS treatment group received 0.22 g zinc sulfate monohydrate (equivalent to 80 mg of elemental zinc) (Sigma-Aldrich Company, St. Louis, MO) combined with 0.44 g milk replacer powder. The placebo treatment group received approximately 0.44 g fresh milk replacer powder. Zinc supplementation was based on a previously published clinical trial, toxicological studies, and nutritional guidelines [11, 22–25]. The milk replacer powder used in treatment preparation was the same product used in the pre-weaned calf milk ration. Treatments were weighed (GX-2000 precision scale; A&D Co Ltd., San Jose, CA) at the Dairy Epidemiology Laboratory at the University of California, Davis Veterinary Medicine Teaching and Research Center (VMTRC; Aly Lab) in Tulare, CA and placed in 2.0 mL microcentrifuge tubes with polypropylene snap caps (Fisher Scientific, Pittsburgh, PA). Prior to study commencement, a color was randomly and permanently assigned to each treatment group, after which treatment tubes, calf milk bottles and calf hutches were marked with either pink, orange, or yellow ink. The study investigators and technicians responsible for treatment preparation, allocation, administration and data collection were blinded to the color assignment until completion of the trial.

### Data collection

For each calf, the study period started at enrollment (24 to 48 hours of age) and ended when the calf exited the hutch (approximately 90 days of age) and from here onwards will be referred to as the “pre-weaning period.” Calf enrollment and study procedures were performed daily at the time of morning feeding. At enrollment, calf characteristics, including sex, birth date, time of first colostrum feeding, and treatment color were recorded. Attitude and feces were assessed daily until 28 days post-enrollment using previously published methods [11] by two study investigators, a veterinarian and a trained researcher. Attitude scoring was based on a three-point scale. A calf with an attitude score of 1 was bright, alert, and readily stood with stimulation; a calf with a score of 2 was quiet, alert, and stood only with moderate stimulation; a calf with a score of 3 exhibited a dull mentation and remained recumbent in response to stimulation. Fecal scoring was performed only on fresh feces and was also based on a three-point scale, as 1 (solid), 2 (semi-formed/loose), or 3 (watery). If no fresh feces were observed in the hutch, “none seen” (NS) was recorded. Body weight was measured using a digital scale at birth, end of treatment, and hutch exit by farm employees with the exception of end of treatment weights, which were recorded by study investigators. Treatments for farm-diagnosed illnesses were performed and recorded on hutch cards by the calf manager. Study investigators regularly recorded this information from cards in addition to extracting treatment event reports from DairyComp 305 (Valley Agricultural Software, Tulare, CA). Though daily diagnosis and treatment of study calves was performed and recorded by the calf manager, a veterinarian was responsible for examining and determining whether study calves met specific criteria for euthanasia. A calf was euthanized if morbidity was severe enough to significantly depress appetite, hydration status, attitude, mentation, and/or ambulatory capability and the calf showed limited to no immediate response to therapy or supportive care. Study calves were euthanized by the calf manager using an on-farm captive bolt protocol established by the herd veterinarian within 3 hours of the decision to euthanize. Enrolled calves that died prior to hutch exit were necropsied within 24 hours of death by a veterinarian. All calves were monitored throughout the study period for evidence of zinc toxicity. At the end of the study period, calves were cared for at the dairy in accordance with standard commercial operations.

Using the same random number generator, a random sample of 127 calves was selected for additional biologic sampling. Approximately 8 to 10% of the study population was selected due to the financial constraints of additional laboratory testing. Serum zinc concentration at baseline and in response to treatment were evaluated for the three treatment groups. Feces collected on the first day of diarrhea and at diarrhea resolution were evaluated for four fecal pathogens (*Escherichia*. *coli* K99, bovine rotavirus and coronavirus, and *Cryptosporidium parvum* oocysts).

### Sample analysis

#### Serum total protein

At enrollment, blood from each calf was collected from the jugular vein using a 20 gauge 1-inch multi-sample needle (Exelint International Co., Redondo Beach, CA) and placed into a 10 mL red top serum tube (BD Vacutainer, Franklin Lakes, NJ) for determination of total protein. Samples remained at room temperature for up to 12 hours until clotting and were then centrifuged (International Equipment Company, CRU-5000, Needham Heights, MA) for 15 minutes. Total protein (g/dL) was measured by a single investigator (HF) on decanted serum using a handheld refractometer (Sper Scientific, Model 300005, Scottsdale, AZ).

#### Serum zinc

For the 127 randomly-sampled calves, additional blood was collected at enrollment and on the last day of treatment, as described above, and placed into 6.0 mL trace element tubes (BD Vacutainer, Franklin Lakes, NJ). Serum was extracted, as described above, and placed in a 2.0 mL microcentrifuge tube with a polypropylene snap cap (Fisher Scientific, Pittsburgh, PA) and stored at −20°C until analysis. Using the same random number generator, 36 of the 127 sampled calves were randomly selected for analysis due to limited financial resources. The pre- and post-zinc supplementation serum samples from each calf (n = 72) were analyzed for zinc concentration (ppm) by ICP-OES (inductively coupled plasma–optical emission spectroscopy) at the CAHFS Laboratory. Quality control samples, including method blanks, laboratory control spikes, and reference Sigma serum, were run with each set of study samples.

#### Fecal analysis

For 127 randomly-sampled calves at the first diarrhea episode, fecal samples were collected at two time points, the first day of diarrhea (fecal score > 1) and the day diarrhea resolved (second day of fecal score = 1). Using new gloves and sterile lubricant, fresh feces was collected by digital rectal stimulation into 20 mL polypropylene twist-top jars (The Cary Company, Addison, IL) and stored at -20°C until analysis. Fecal samples were tested at the Dairy Epidemiology Laboratory, VMTRC by a veterinarian for *E*. *coli* K99, bovine rotavirus and coronavirus, and *C*. *parvum* oocysts using a commercial kit (Pathasure Enteritis 4; Biovet, Quebec, Canada) that is highly specific (> 90%) and sensitive (*E*. *coli* K99, 93%; rotavirus, 100%; coronavirus, 77%) [26, 27]. For calves with a first-day diarrhea sample on or before 7 days of age, both fecal samples were tested for all four pathogens. For calves with a first-day diarrhea sample after 7 days of age, both fecal samples were tested for three pathogens (*C*. *parvum*, bovine rotavirus and coronavirus). Samples from calves older than 7 days of age were not tested for *E*. *coli* K99 based on calves’ susceptibility [28]. Testing was performed according to kit manufacturer guidelines, and a low-temperature incubator (Fisher Scientific, Model 146, Pittsburgh, PA) was used during incubation periods. Test results were recorded as positive or negative using control wells for color comparison. If the color change was darker than the negative control, the sample was considered positive.

#### Milk zinc

For each of the 107 study days (December 15, 2015 to March 31, 2016) of zinc supplementation, approximately 1.5 mL of treated milk from two bottles of each treatment group were randomly collected into 2.0 mL microcentrifuge tubes with polypropylene snap caps (Fisher Scientific, Pittsburgh, PA), and stored at −20°C until analysis. At the time of analysis, milk samples were thawed at 4°C, vortexed, pooled by week and treatment group, and analyzed for zinc concentration (ppm) by ICP-MS (inductively coupled plasma mass spectrometry) at the CAHFS Laboratory. Quality control samples, including method blanks, laboratory control spikes, National Institute of Standards and Technology (NIST) reference materials (NIST 1640), and a spiked milk sample, were run with each set of study samples.

### Statistical analyses

Data analysis was performed using R Statistic Software version 3.3.1 and Stata IC 14.2 (College Station, TX). Statistical differences were determined at the 5% level of significance using per protocol analysis. An ANOVA was used to compare calves in each treatment group at enrollment with respect to birth weight (kg), serum total protein (g/dL), attitude score, and fecal score. Oral zinc dose at the start and end of treatment was calculated as the zinc supplementation dose (80 mg) divided by calf body weight (kg) at birth and on the last day of treatment, respectively. An ANOVA was used to compare oral zinc dose (mg/kg) at treatment start and end as well as mean body weight (kg) at birth, end of treatment, and hutch exit between treatment groups and between bulls and heifers. A Chi-Square test of Independence was used to compare the proportions of calves by sex as well as mortality between treatment groups. For all analyses, Tukey’s Honest Significant Difference method was used to generate pairwise comparisons to further characterize significant differences identified by ANOVA. Residual diagnostics, including Residuals vs. Fitted, Scale-Location, Normal Q-Q, and Cook's distances plots, were used to validate all ANOVA model assumptions. The non-parametric Kruskal-Wallis Rank Sum test was used when assumptions were violated.

For the randomly-sampled calves, Fisher exact tests were used to compare fecal pathogen prevalence on the first day of diarrhea and at diarrhea resolution between treatment groups. Pairwise comparisons with Bonferroni adjustment were used to identify specific differences in pathogen prevalence. An ANOVA was used to compare serum zinc concentration before and after treatment between treatment groups and between bulls and heifers. A Kruskal-Wallis Rank Sum test was used to compare rank sums of zinc concentrations in pooled milk samples from different treatment groups. Post-hoc Nemenyi-tests for pairwise multiple comparisons of ranked data were used to identify specific differences in zinc concentrations between groups.

For all regression models in this study, univariate regression was first used to evaluate associations between individual predictor variables and outcomes. All variables with statistical and/or biological significance were initially included in multivariate regression models. The final models were built using a manual backwards elimination procedure, with a significance level of P > 0.05 as the removal criterion. Confounding was assessed using the method of change of estimates, where a 10% or greater change in the estimate of the treatment group regression coefficient between the models with and without the confounder variable was used as evidence of confounding [29–32]. Variables identified as confounders were included in the final model. All possible interactions between treatment group and predictor variables were explored and retained in the final model if statistically significant.

#### Microbiological cure

For the randomly-sampled calves, logistic regression was used to evaluate associations between microbiological cure and treatment group. Other predictor variables of interest included sex, serum total protein, and age on the first day of diarrhea. Microbiological cure was defined as a negative fecal ELISA test at resolution of clinical diarrhea for calves with a positive ELISA test on the first day of diarrhea for at least one of the four fecal pathogens (*E*. *coli* K99, bovine rotavirus and coronavirus, and *C*. *parvum*). Models were generated for each fecal pathogen individually and an overall model, which evaluated microbiological cure at clinical diarrhea resolution for calves that tested positive for any single pathogen on the first day of diarrhea. Serum total protein and calf age at first diarrhea were included in all final models to control for potential confounding by passive transfer status and age.

#### Mean daily weight change

Linear regression was used to evaluate associations between ADG (kg) and treatment group during the treatment and pre-weaning periods separately. Other predictor variables of interest included sex, birth weight (kg), serum total protein, number of days having diarrhea, age, and volume (L) of milk, Calva, and NuLife electrolytes fed at either end of treatment or hutch exit. For each calf, ADG during the treatment and pre-weaning period was calculated as the difference between birth and end treatment or hutch exit weight, respectively, divided by the number of days between these time points. To explore the possibility of an interaction between treatment group, sex, and birth weight, the final linear regression model for ADG during the pre-weaning period was stratified by sex. Age at the end of treatment or hutch exit and number of days with diarrhea were dropped from all final models in favor of improved Akaike information criterion (AIC).

#### Onset of diarrhea and clinical cure

For all survival analyses, diarrhea was defined as a fecal score greater than 1 while diarrhea cure was defined as the second consecutive day of normal feces (fecal score of 1) following the first diarrhea episode. Subsequent episodes of diarrhea were not included in the analysis. Calves that died or did not experience diarrhea or cure from diarrhea were censored. If fresh feces were not observed on daily calf hutch assessment, a fecal score was not recorded for that day and not included in the analyses. Kaplan-Meier analysis was used to determine median days to first diarrhea event and, for those calves that developed diarrhea during the assessment period, median days to clinical diarrhea cure. A Log Rank test of equality was used to compare survivor functions between treatments.

Cox Proportional Hazards regression analysis was used to estimate and compare the hazard of diarrhea and diarrhea cure between treatment groups. Sex, age, serum total protein at enrollment, birth weight (kg), antimicrobial therapy, and application of fresh straw to the hutches were evaluated as predictor variables and potential confounders. When modeling the hazard of diarrhea cure, a binary variable termed therapeutic supplementation indicating whether calves were treated with either ZM, ZS or placebo for all or at least the first 5 days of diarrhea was evaluated as an additional covariate. A five-day period was selected by the authors based on clinical experience, as five days represents a reasonable duration over which most therapeutic treatments for calf diarrhea should be applied and be expected to alleviate disease. The proportional hazards assumption that the hazard of diarrhea is independent of time was assessed using analysis of Schoenfeld residuals and testing whether the log hazard-ratio function is constant over time. Any variable found to violate the proportional hazards assumption was included in the final regression model as a time varying covariate.

## Results

### Enrollment and baseline comparisons

A total of 1,513 calves were enrolled in the trial. However, due to failure to immediately recognize exclusion criteria, 23 calves were excluded shortly after enrollment. In addition, 8 calves were excluded due to treatment errors. Therefore, a total of 1,482 calves (placebo = 500, ZM = 491, ZS = 491) were included in the final analyses. A total of 242 calves (16.3%) had minimal fecal output at the time of enrollment while 125 calves (8.4%) had abnormal fecal scores of 2 or 3 that were described as meconium. All enrolled calves appeared healthy on visual assessment and hence were assumed to have a normal fecal score at the time of enrollment. The three treatment groups at enrollment did not differ significantly in mean birth weight (kg) (*P* = 0.244), mean serum total protein (g/dL) (*P* = 0.541), mean attitude score (*P* = 0.845), mean fecal score (*P* = 0.522), as shown in Table 1, or distribution of calf sex (*P* = 0.472). Of the 1,482 study calves, 21 (1.4%) died during the trial: 5 calves in the placebo group (1.0%), 11 calves in the ZM group (2.2%), and 5 calves in the ZS group (1.0%). Of these 21 calves that died, 14 (66.7%) were bulls and 7 (33.3%) were heifers. Eighteen of the 21 calves (85.7%) were found dead, rather than euthanized, due to acute and spontaneous death without previous obvious clinical signs of disease. The remaining 3 calves were euthanized prior to death due to severe and/or prolonged morbidity. Characteristics and causes of death based on field necropsy of these calves can be found in S4 Table. There was no significant difference in the proportion of calves that died between treatment groups (*P* = 0.168), though mortality was significantly higher in bulls compared to heifer calves (*P* = 0.049). Birth weight data were available for all 1,482 calves. Due to calf mortality between enrollment and completion of treatment (n = 4), end treatment weight data were available for 1,478 calves. Similarly, due to calf mortality (n = 21) or missing data for body weight at hutch exit from the dairy’s records (n = 40), hutch exit weight data were available for 1,421 calves. A summary of body weight data stratified by treatment group and sex is presented in Table 2. Within each treatment group, bull calves showed consistently higher birth weight (P < 0.001), end treatment weight (P < 0.001), and exit hutch weight (P < 0.001) compared to heifer calves. However, at birth, end of treatment, or hutch exit there were no differences in body weight between treatment groups for bulls (*P* > 0.1) or heifers (*P* > 0.1). The mean attitude scores during the study period were 1.2 across the three treatment groups (*P* = 0.208). Of 1,482 calves included in the final analysis, 475 (95.0%) Placebo, 458 (93.3%) ZM, and 461 (93.9%) ZS calves acquired diarrhea. For diarrheic calves, the mean fecal score was 1.4 across all treatment groups and the mean diarrhea duration was 7.4, 6.8, and 6.9 days for Placebo, ZM, and ZS treated calves, respectively.

**Table 1:** Comparison of continuous baseline traits of neonatal Holstein calves (n = 1,482) at enrollment prior to treatment administration by treatment group using ANOVA from a double-blind block-randomized clinical trial.

Variable	Treatment^1^^,^^2^														
	Placebo (n = 500)					Zinc methionine (n = 491)					Zinc sulfate (n = 491)				
	n	Mean	SE	95% CI		n	Mean	SE	95% CI		n	Mean	SE	95% CI	
				Lower	Upper				Lower	Upper				Lower	Upper
Birth WT^3^	500	40.95	0.255	40.45	41.45	491	40.54	0.256	40.04	41.05	491	41.15	0.267	40.63	41.67
Serum TP^4^	500	6.35	0.028	6.30	6.41	491	6.33	0.028	6.27	6.38	491	6.37	0.026	6.32	6.42
Attitude score^5^	500	1.31	0.021	1.27	1.35	491	1.29	0.020	1.25	1.33	491	1.30	0.021	1.26	1.34
Fecal score^6^	414	1.09	0.016	1.06	1.12	415	1.12	0.017	1.09	1.15	411	1.11	0.017	1.08	1.14

^1^Treatments: placebo = 0.44 g fresh milk replacer powder (MRP); zinc methionine = 80 mg of zinc (0.45 g zinc methionine complex as Zinpro180) in 0.44 g of fresh MRP; zinc sulfate = 80 mg of zinc (0.22 g zinc sulfate monohydrate) in 0.44 g of fresh MRP.

^2^No significant differences in mean birth weight (P = 0.244), mean serum total protein (P = 0.541), mean attitude score (P = 0.845), or mean fecal score (P = 0.522) between treatment groups using ANOVA.

^3^Birth WT = birth weight (kg).

^4^Serum TP = serum total protein (g/dL).

^5^Attitude scores: 1 = calf was bright, alert, and readily stood with stimulation; 2 = calf was quiet, alert, and stood only with moderate stimulation; 3 = calf was recumbent with little or no response to stimulation.

^6^Fecal scores: 1 = solid; 2 = semi-formed/loose; 3 = watery. Baseline comparisons of fecal score excluded calves whose feces was “not seen” at enrollment: placebo (n = 86), zinc methionine (n = 76), zinc sulfate (n = 80).

**Table 2:** Comparison of mean crude body weight at birth, end of treatment, and exit from the hutch of neonatal Holstein calves (n = 1,482) by treatment group and sex using ANOVA from a double-blind block-randomized clinical trial.

Placebo^1^	Bulls					Heifers				
	n^4^	Mean	SE	95% CI		n^4^	Mean	SE	95% CI	
				Lower	Upper				Lower	Upper
Birth weight (kg)	216	43.39^a^	0.374	42.66	44.13	284	39.09^b^	0.305	38.49	39.69
End treatment weight (kg)	215	46.17^c^	0.336	45.52	46.83	284	41.92^d^	0.287	41.36	42.48
Exit hutch weight (kg)	208	105.48^e^	0.813	103.89	107.08	274	101.40^f^	0.670	100.09	102.71
Zinc methionine^2^	Bulls					Heifers				
	n^4^	Mean	SE	95% CI		n^4^	Mean	SE	95% CI	
				Lower	Upper				Lower	Upper
Birth weight (kg)	202	43.27^a^	0.389	42.51	44.04	289	38.64^b^	0.291	38.06	39.21
End treatment weight (kg)	202	45.94^c^	0.355	45.25	46.64	287	41.63^d^	0.265	41.11	42.15
Exit hutch weight (kg)	191	107.56^e^	0.823	105.94	109.17	274	99.55^f^	0.728	98.12	100.97
Zinc sulfate^3^	Bulls					Heifers				
	n^4^	Mean	SE	95% CI		n^4^	Mean	SE	95% CI	
				Lower	Upper				Lower	Upper
Birth weight (kg)	221	44.00^a^	0.369	43.28	44.73	270	38.82^b^	0.316	38.20	39.43
End treatment weight (kg)	221	46.65^c^	0.320	46.02	47.28	269	41.68^d^	0.300	41.10	42.27
Exit hutch weight (kg)	214	105.87^e^	0.762	104.37	107.36	260	100.12^f^	0.755	98.64	101.60

^a-f^Means with different superscripts within rows and columns are significantly different (P < 0.05) according to ANOVA.

^1^Placebo = 0.44 g fresh milk replacer powder (MRP).

^2^Zinc methionine = 80 mg of zinc (0.45 g zinc methionine complex as Zinpro180) in 0.44 g of fresh MRP.

^3^Zinc sulfate = 80 mg of zinc (0.22 g zinc sulfate monohydrate) in 0.44 g of fresh MRP.

^4^Changes in n were due to calf mortality, severe morbidity, or missing body weight data.

### Milk and serum zinc

A total of 629 treated milk samples were obtained throughout the 107-day study period and pooled by treatment group and week, yielding a total of 16 pooled samples per treatment group. Zinc concentrations (ppm) were significantly higher in pooled milk samples treated with ZM (*P* < 0.001) and ZS (*P* < 0.001) compared to placebo-treated samples, and there were no significant differences between ZM and ZS-treated samples (*P* = 1.000), as shown in S5 Table.

Within zinc treatment groups, oral zinc dose at the start and end of treatment is summarized by sex in S6 Table. For both ZM- and ZS-treated calves, oral zinc dose at the start (*P* < 0.001) and end (*P* < 0.001) of treatment was significantly higher in heifer versus bull calves. Serum zinc concentrations before and after treatment were obtained from 36 calves (n = 12 for each treatment group) and are summarized in S7 Table. Overall, there were no significant differences in mean pre-treatment serum zinc concentrations between treatment groups (*P* = 0.233). Mean post-treatment serum zinc concentrations were significantly higher in calves treated with ZM (*P* < 0.001) and ZS (*P* = 0.002) compared to placebo-treated calves, and there were no significant differences among calves treated with ZM and ZS (*P* = 0.406). Stratification of serum zinc data by treatment group and sex demonstrated that for ZM-treated calves, heifers had a numerically higher post-treatment serum zinc concentration compared to bulls, though the difference was not statistically significant (*P* = 0.199). In contrast, in ZS-treated calves, heifers had a numerically lower post-treatment serum zinc concentration compared to bulls, though the difference was also not statistically significant (*P* = 0.538).

### Fecal analysis

Fecal analysis data were analyzed for 92 of the 127 randomly-selected calves. The remaining 35 calves were not included in the analysis due to not acquiring diarrhea during the assessment period (n = 10), death prior to final sampling (n = 1), exclusion due to improper treatment regimen (n = 1), or incorrect sampling day (n = 23).The 92 calves had a mean age at onset of diarrhea of 13.3, 11.0 and 11.3 days for ZM, ZS and placebo-treated groups, respectively; and a mean age at resolution of diarrhea of 18.8, 16.1 and 15.7 days for ZM, ZS and placebo-treated groups, respectively. There were no significant differences in the prevalence of *E*. *coli* K99 (*P* = 0.694), rotavirus (*P* = 0.331), coronavirus (*P* = 0.819), or *C*. *parvum* (*P* = 0.719) fecal shedding on the first day of diarrhea between treatment groups (S8 Table). There were no significant differences in the prevalence of *E*. *coli* K99 (*P* = 0.256), rotavirus (*P* = 0.344), or coronavirus (*P* = 1.000) fecal shedding at resolution of diarrhea between treatment groups, though there was a difference in *C*. *parvum* (*P* = 0.006) fecal shedding between treatment groups (S9 Table). The prevalence of *C*. *parvum* fecal shedding at resolution of diarrhea was significantly higher in calves treated with ZM (*P* = 0.009) and ZS (*P* = 0.023) compared to placebo-treated calves, and there were no significant differences among calves treated with ZM and ZS (*P* = 1.000).

### Microbiological cure

Results of logistic regression models for overall and pathogen-specific microbiological cure are presented in Tables 3–5. For pathogen-specific cure, all calves that tested positive on the first day of diarrhea for coronavirus (n = 4) tested negative at clinical diarrhea resolution. For calves that tested positive on the first day of diarrhea for *E*. *coli* K99 (n = 9), all placebo-treated calves (n = 2) tested negative at clinical diarrhea resolution while half (n = 2) of all ZS-treated calves tested either positive or negative at clinical diarrhea resolution, resulting in omission of ZS treatment variable from the model due to collinearity. Hence, logistic regression analyses for microbiological cure in calves that tested positive for coronavirus and *E*. *coli* K99 were not possible. For 59 calves that tested positive for rotavirus on the first day of diarrhea (Table 3), calves treated with ZM had a 50% increased odds of testing negative at diarrhea resolution compared to placebo-treated calves, though this difference was not significant (*P* = 0.549). Likewise, calves treated with ZS had 100% increased odds (2 times the odds) of testing negative for rotavirus at diarrhea resolution compared to placebo-treated calves, though this difference was also not significant (*P* = 0.314). However, this model demonstrated a significant main effect of serum total protein, such that for every 1 unit (g/dL) increase in serum total protein at enrollment, the odds of microbiological cure of rotavirus decreased by 79% (*P* = 0.026). For 40 calves that tested positive for *Cryptosporidium parvum* on the first day of diarrhea (Table 4), calves treated with ZM had an 87% reduced odds of testing negative at diarrhea resolution compared to placebo-treated calves, though this difference was not significant (*P* = 0.119). Likewise, calves treated with ZS had a 74% reduced odds of testing negative for *Cryptosporidium parvum* at diarrhea resolution compared to placebo-treated calves, though this difference was also not significant (*P* = 0.183). For 55 calves that tested positive for any one of the four fecal pathogens (*E*. *coli* K99, rotavirus, coronavirus, *Cryptosporidium parvum*) on the first day of diarrhea (Table 5), calves treated with ZM had a 25% increased odds of testing negative at diarrhea resolution compared to placebo-treated calves, though this difference was not significant (*P* = 0.769). Likewise, calves treated with ZS had a 52% increased odds of testing negative for *Cryptosporidium parvum* at diarrhea resolution compared to placebo-treated calves, though this difference was also not significant (*P* = 0.633). However, this model demonstrated that heifer calves had 71% lower odds of microbiological cure of any single fecal pathogen compared to bull calves (*P* = 0.076).

**Table 3:** Final logistic regression model from a double-blind block-randomized clinical trial on the effect of treatment with zinc methionine or zinc sulfate compared to placebo on microbiological cure^1^ of rotavirus in neonatal Holstein calves (n = 59).

Variable	β	Odds ratio	SE (OR)	95% CI (OR)		*P*-value
				Lower	Upper	
Treatment^2^						
Placebo	Reference					
Zinc methionine	0.417	1.518	1.056	0.388	5.936	0.549
Zinc sulfate	0.717	2.048	1.458	0.508	8.263	0.314
Serum total protein^3^	-1.269	0.281	0.160	0.092	0.857	0.026
Calf age at first diarrhea	0.208	1.231	0.112	1.030	1.471	0.022

^1^Microbiological cure is defined as a negative fecal ELISA test (Pathasure Enteritis 4; Biovet, Quebec, Canada) for rotavirus at clinical diarrhea resolution for calves with a positive fecal ELISA test for rotavirus on the first day of diarrhea.

^2^Treatments: placebo = 0.44 g fresh milk replacer powder (MRP; zinc methionine = 80 mg of zinc (0.45 g zinc methionine complex as Zinpro180) in 0.44 g of fresh MRP; zinc sulfate = 80 mg of zinc (0.22 g zinc sulfate monohydrate) in 0.44 g of fresh MRP.

^3^Serum total protein (g/dL) was measured between 24 and 48 hours of age by handheld refractometry.

**Table 4:** Final logistic regression model from a double-blind block-randomized clinical trial on the effect of treatment with zinc methionine or zinc sulfate compared to placebo on microbiological cure^1^ of *Cryptosporidium parvum* at resolution of clinical diarrhea in neonatal Holstein calves positive for *Cryptosporidium parvum* on the first day of clinical diarrhea (n = 40).

Variable	β	Odds ratio	SE (OR)	95% CI (OR)		*P*-value
				Lower	Upper	
Treatment^2^						
Placebo	Reference					
Zinc methionine	-2.047	0.129	0.169	0.010	1.689	0.119
Zinc sulfate	-1.350	0.259	0.263	0.036	1.890	0.183
Serum total protein^3^	-0.967	0.380	0.310	0.077	1.879	0.235
Calf age at first diarrhea	-0.185	0.831	0.072	0.701	0.985	0.033

^1^Microbiological cure is defined as a negative fecal ELISA test (Pathasure Enteritis 4; Biovet, Quebec, Canada) for Cryptosporidium parvum at clinical diarrhea resolution for calves that had a positive fecal ELISA test for Cryptosporidium parvum on the first day of diarrhea.

^2^Treatments: placebo = 0.44 g fresh milk replacer powder (MRP); zinc methionine = 80 mg of zinc (0.45 g zinc methionine complex as Zinpro180) in 0.44 g of fresh MRP; zinc sulfate = 80 mg of zinc (0.22 g zinc sulfate monohydrate) in 0.44 g of fresh MRP.

^3^Serum total protein (g/dL) was measured between 24 and 48 hours of age by handheld refractometry.

**Table 5:** Final logistic regression model from a double-blind block-randomized clinical trial on the effect of treatment with zinc methionine or zinc sulfate compared to placebo on microbiological cure at resolution of clinical diarrhea for calves that tested positive for any single pathogen (*E*. *coli* K99, rotavirus, coronavirus, *Cryptosporidium parvum*) on the first day of diarrhea^1^ (n = 55).

Variable	β	Odds ratio	SE (OR)	95% CI (OR)		*P*-value
				Lower	Upper	
Treatment^2^						
Placebo	Reference					
Zinc methionine	0.226	1.253	0.962	0.278	5.643	0.769
Zinc sulfate	0.419	1.521	1.334	0.272	8.490	0.633
Sex						
Bull	Reference					
Heifer	-1.223	0.293	0.203	0.075	1.139	0.076
Serum total protein^3^	-0.881	0.414	0.258	0.122	1.406	0.158
Calf age at first diarrhea	-0.131	0.877	0.058	0.770	0.999	0.049

^1^Microbiological cure is defined as a negative fecal ELISA test (Pathasure Enteritis 4; Biovet, Quebec, Canada) at clinical diarrhea resolution for calves that had a positive fecal ELISA test for any single pathogen on the first day of diarrhea.

^2^Treatments: placebo = 0.44 g fresh milk replacer powder (MRP); zinc methionine = 80 mg of zinc (0.45 g zinc methionine complex as Zinpro180) in 0.44 g of fresh MRP; zinc sulfate = 80 mg of zinc (0.22 g zinc sulfate monohydrate) in 0.44 g of fresh MRP.

^3^Serum total protein (g/dL) was measured between 24 and 48 hours of age by handheld refractometry.

### Average daily gain

A total of 1,482 calves were included in the linear regression model results for ADG during the treatment period, which are presented in Table 6. There was no significant difference in ADG for ZM- or ZS-treated calves compared to placebo-treated calves, though there were significant main effects of sex, birth weight, and milk volume. Specifically, heifer calves gained 70 g bodyweight per day less compared to bull calves (*P* < 0.001). For every 1 kg increase in birth weight, calves gained 16 g per day less than their herd mates (*P* < 0.001). For every 1 L increase in milk volume per day during the treatment period, calves gained an additional 13 g per day (*P* < 0.001).

**Table 6:** Final linear regression model from a double-blind block-randomized clinical trial on the effect of treatment with zinc methionine (ZM) or zinc sulfate (ZS) compared to placebo on average daily gain in kilograms in neonatal Holstein calves (n = 1,482) during the treatment period.

Variable	β	SE	95% CI		*P*-value
			Lower	Upper	
Treatment^1^					
Placebo	Reference				
Zinc methionine	-0.008	0.014	0.035	0.019	0.565
Zinc sulfate	-0.000	0.014	-0.028	0.027	0.983
Sex					
Bull	Reference				
Heifer	-0.070	0.013	-0.094	0.045	0.000
Birth weight (kg)	-0.016	0.001	-0.018	-0.014	0.000
Milk volume^2^	0.013	0.002	0.009	0.016	0.000
Intercept	0.164	0.126	-0.083	0.410	0.193

^1^Treatments: placebo = 0.44 g fresh milk replacer powder (MRP); zinc methionine = 80 mg of zinc (0.45 g zinc methionine complex as Zinpro180) in 0.44 g of fresh MRP; zinc sulfate = 80 mg of zinc (0.22 g zinc sulfate monohydrate) in 0.44 g of fresh MRP.

^2^Total milk volume (L) fed to calves during the treatment period.

Table 7 summarizes the linear regression analysis of ADG during the pre-weaning period for 1,421 calves which showed a significant difference in ADG for ZM-treated calves compared to placebo-treated calves and in bull versus heifer calves. Milk volume had a significant effect on ADG, such that for every 1 L increase in milk volume per day during the treatment period, calves gained an additional 2 g bodyweight per day (*P* = 0.001). Results of the final model showed a significant main effect for ZM-treated bull calves and a significant interaction term for ZM treatment by sex. After controlling for milk volume received during the treatment period, ZM-treated bulls gained 22 g body weight per day on average more than placebo-treated bull calves (*P* = 0.042) and ZM-treated heifers gained 12 g less body weight per day on average compared to placebo-treated heifers (*P* = 0.019). When considering the model coefficients for treatment group, sex, and their interaction, bull calves treated with ZM gained 454 g per day (0.432 + 0.022) while female calves treated with ZM gained 0.404 g per day (0.432 + 0.022–0.016–0.034), hence 50 g per day more gain in male calves compared to heifers treated with ZM (*P* = 0.019). For ZS-treated calves, there was a numerical decrease in weight gain of 5 g per day in bulls and 11 g per day in heifers compared to placebo-treated calves, though the differences were not significant (*P* = 0.673 bulls; *P* = 0.681 heifers).

**Table 7:** Final linear regression model from a double-blind block-randomized clinical trial on the effect of treatment with zinc methionine (ZM) or zinc sulfate (ZS) compared to placebo on average daily gain in kilograms in neonatal Holstein calves (n = 1,421) during the pre-weaning period.

Variable	β	SE	95% CI		*P*-value
			Lower	Upper	
Treatment^1^					
Placebo	Reference				
Zinc methionine	0.022	0.011	0.001	0.044	0.042
Zinc sulfate	-0.005	0.011	-0.025	0.016	0.673
Sex					
Bull	Reference				
Heifer	-0.016	0.010	-0.035	0.004	0.120
Milk volume^2^	0.002	0.001	0.001	0.003	0.001
ZM x Heifer	-0.034	0.014	-0.062	-0.006	0.019
ZS x Heifer	-0.006	0.014	-0.034	0.022	0.681
Intercept	0.432	0.082	0.271	0.593	0.000

^1^Treatments: placebo = 0.44 g fresh milk replacer powder (MRP); zinc methionine = 80 mg of zinc (0.45 g zinc methionine complex as Zinpro180) in 0.44 g of fresh MRP; zinc sulfate = 80 mg of zinc (0.22 g zinc sulfate monohydrate) in 0.44 g of fresh MRP.

^2^Total milk volume (L) fed to calves during the assessment period.

Linear regression models of ADG during the pre-weaning period were stratified by sex in order to avoid interpreting a three-way interaction between treatment group, sex, and birth weight. In the heifer model (S10 Table), the interaction between ZM treatment and birth weight was significant which implied that birth weight modified the effect of ZM treatment on ADG. At a 29 kg birth weight (two standard deviations below the mean), ZM-treated heifers gained 49 g body weight per day on average less than placebo-treated heifers (*P* = 0.037). However, at a 49 kg birth weight (two standard deviations above the mean), ZM-treated heifers gained 30 g body weight per day on average more than placebo-treated heifers (*P* = 0.037). In the bull calves model (S11 Table) there was no significant interaction between treatment group and birth weight.

### Onset of diarrhea and clinical cure

**Fig 1:**
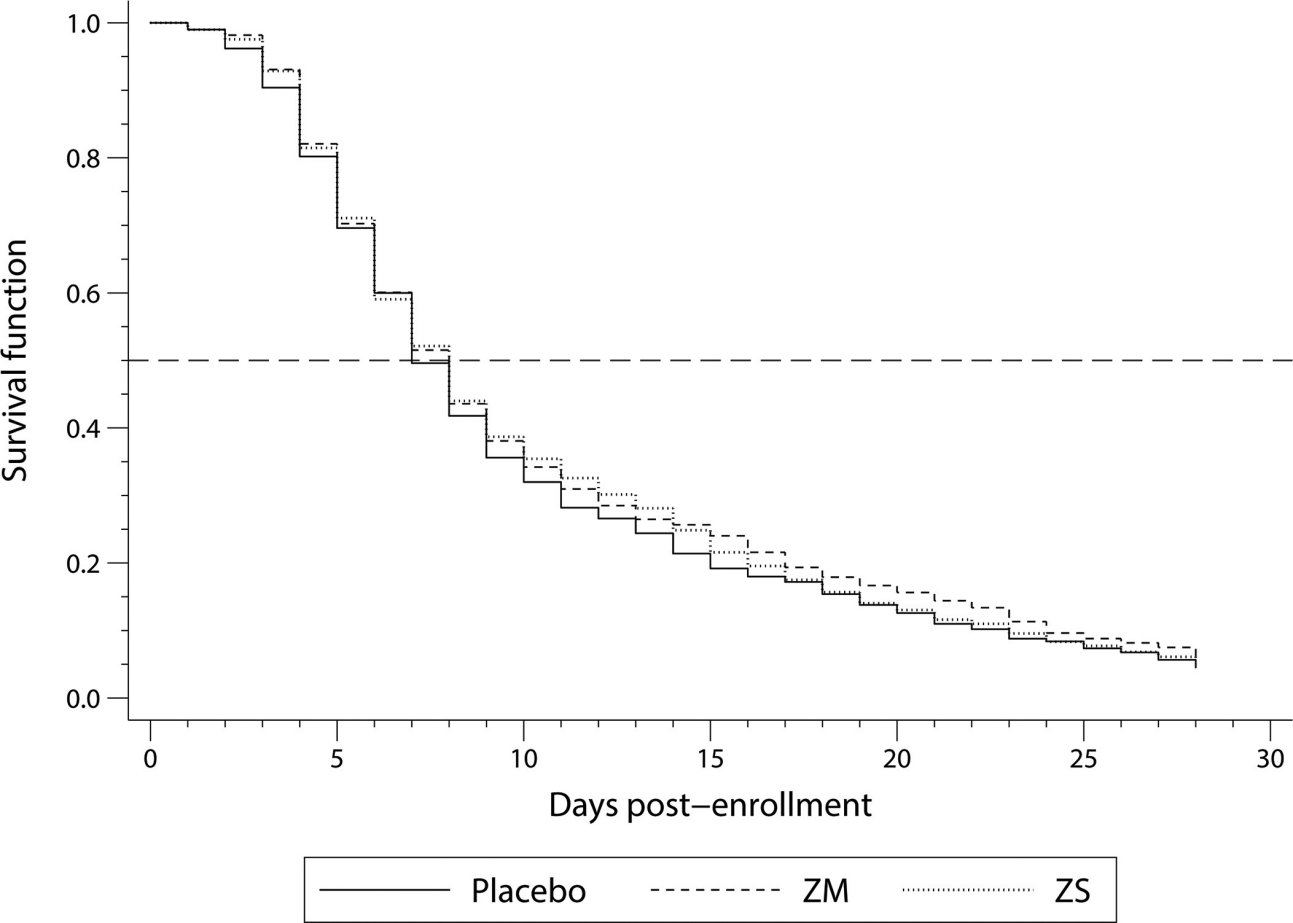
Graph of the Kaplan-Meier survival function of days to first diarrhea in neonatal Holstein calves (n = 1,482) for three different treatment groups from a double-blind block-randomized clinical trial. Treatments included: 1) placebo = 0.44 g fresh milk replacer powder (MRP) (n = 500); 2) zinc methionine (ZM) = 80 mg of zinc (0.45 g zinc methionine complex as Zinpro180) in 0.44 g of fresh MRP (n = 491); 3) zinc sulfate (ZS) = 80 mg of zinc (0.22 g zinc sulfate monohydrate) in 0.44 g of fresh MRP (n = 491).

A total of 1,482 calves were included in the Kaplan-Meier survival analysis of time to first diarrhea event (Fig 1). There were no significant differences in median age at onset of diarrhea, specifically, 8, 8 and 7 days for the ZM, ZS and placebo-treated calves, respectively (*P* = 0.402). Cox proportional hazard regression model for diarrhea hazard are presented in Table 8. After controlling for age, calves treated with ZM had a 14.7% reduced hazard of diarrhea compared to placebo-treated calves (*P* = 0.015). Calves treated with ZS had 13.9% reduced hazard of diarrhea compared to placebo-treated calves (*P* = 0.022).

**Table 8:** Final Cox proportional hazard regression model from a double-blind block-randomized clinical trial on the effect of treatment with zinc methionine or zinc sulfate compared to placebo on days to first diarrhea^1^ event in neonatal Holstein calves (n = 1,482).

Variable	β	Hazard Ratio (HR)	SE (HR)	95% CI (HR)		*P*-value (HR)
				Lower	Upper	
Treatment^2^						
Placebo	Reference					
Zinc methionine	-0.159	0.853	0.056	0.750	0.970	0.015
Zinc sulfate	-0.150	0.861	0.056	0.757	0.978	0.022
Age (days)	-0.856	0.425	0.016	0.395	0.457	< 0.001

^1^First diarrhea defined as the first day post-enrollment with fecal score greater than 1.

^2^Treatments: placebo = 0.44 g fresh milk replacer powder (MRP); zinc methionine = 80 mg of zinc (0.45 g zinc methionine complex as Zinpro180) in 0.44 g of fresh MRP; zinc sulfate = 80 mg of zinc (0.22 g zinc sulfate monohydrate) in 0.44 g of fresh MRP.

**Fig 2:**
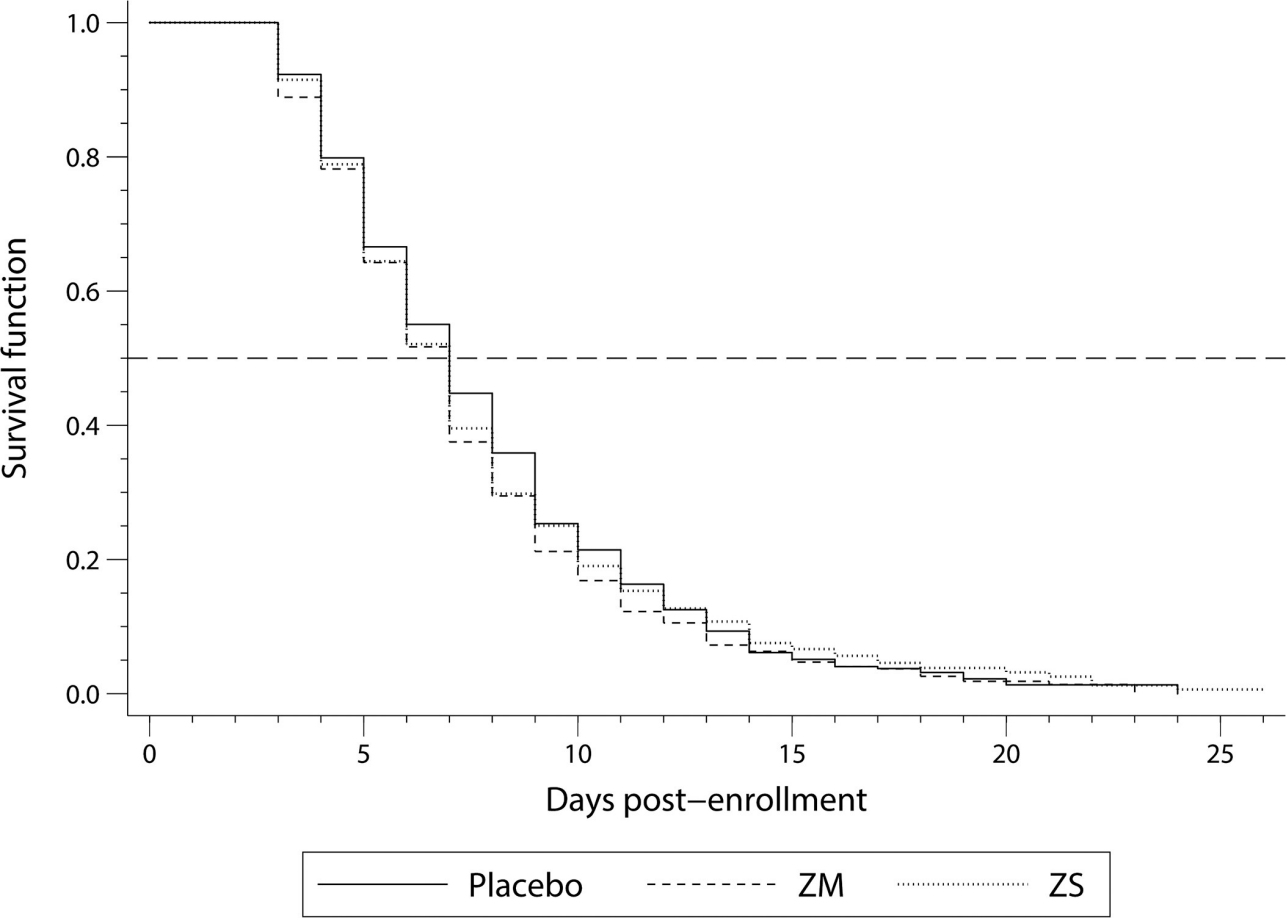
Graph of the Kaplan-Meier survival cure of days to clinical diarrhea cure in neonatal Holstein calves (n = 1,394) for three different treatment groups from a double-blind block-randomized clinical trial. Treatments included: 1) placebo = 0.44 g fresh milk replacer powder (MRP) (n = 500); 2) zinc methionine (ZM) = 80 mg of zinc (0.45 g zinc methionine complex as Zinpro180) in 0.44 g of fresh MRP (n = 491); 3) zinc sulfate (ZS) = 80 mg of zinc (0.22 g zinc sulfate monohydrate) in 0.44 g of fresh MRP (n = 491).

A total of 1,394 calves were included in the Kaplan-Meier survival analysis of time to clinical diarrhea cure (Fig 2), as 88 calves failed to acquire diarrhea during the assessment period. There were no significant differences in the median days to diarrhea cure which was 7 days across all 3 treatment groups (*P* = 0.264). Cox proportional hazard regression model for diarrhea cure hazard are presented in Table 9. Results of the final model showed a significant interaction term between treatment and therapeutic supplementation as well as the need for age as a time varying covariate. When considering calves that did not receive supplementation, respective to each of the 3 groups, for at least the first five days of diarrhea there was no significant difference between either ZM- and ZS-treated calves compared to placebo-treated calves (*P* = 0.223 ZM, *P* = 0.134 ZS). However, when considering calves that were supplemented for at least the first five days of diarrhea, ZM-treated calves experienced a 21.4% higher hazard of cure from diarrhea compared to placebo-treated calves (*P* = 0.027). Likewise, ZS-treated calves experienced a 13.0% higher hazard of cure from diarrhea compared to placebo-treated calves (*P* = 0.040).

**Table 9:** Final Cox proportional hazard regression model from a double-blind block-randomized clinical trial on the effect of treatment with zinc methionine or zinc sulfate compared to placebo on days to clinical diarrhea cure event^1^ in neonatal Holstein calves (n = 1,394).

Variable	β	Hazard Ratio	SE	95% CI (HR)		*P*-value
				Lower	Upper	
Treatment^2^						
Placebo	Reference					
Zinc methionine	-0.190	0.827	0.129	0.609	1.123	0.223
Zinc sulfate	-0.223	0.800	0.119	0.598	1.071	0.134
Therap supp^3^	-0.227	0.797	0.129	0.593	1.071	0.133
Age	0.004	1.004	0.015	0.975	1.034	0.784
ZM x Therap supp	0.384	1.468	0.255	1.044	2.063	0.027
ZS x Therap supp	0.345	1.412	0.237	1.016	1.963	0.040
*Time varying covariate*						
Age	-0.005	0.995	0.002	0.991	0.998	0.004

^1^Clinical diarrhea cure is defined as the second consecutive day of normal feces (fecal score of 1) following the first diarrhea episode.

^2^Treatments: placebo = 0.44 g fresh milk replacer powder (MRP); zinc methionine = 80 mg of zinc (0.45 g zinc methionine complex as Zinpro180) in 0.44 g of fresh MRP; zinc sulfate = 80 mg of zinc (0.22 g zinc sulfate monohydrate) in 0.44 g of fresh MRP.

^3^Therap supp = Therapeutic supplementation, a binary variable indicating whether calves were treated for all or at least the first 5 days of diarrhea.

## Discussion

The current trial demonstrated evidence for the beneficial effect of ZM on ADG and neonatal diarrhea as well as an effect of ZS on diarrhea in dairy calves during the pre-weaning period. It is important to consider these results in the context of the entire pre-weaning and hutch period. On average, after 90 days from birth to hutch exit, placebo-treated bull calves gained 38.88 kg body weight while ZM-treated bull calves gained an additional 1.98 kg (40.86 kg). In contrast, the effect of zinc on weight gain in treated heifers depended on birth weight. Low birth weight heifers treated with ZM gained on average less than a placebo-treated heifer of the same birth weight. In contrast, high birth weight heifers treated with ZM gained more than placebo-treated heifers of the same birth weight. The switch in direction of the association between ZM treatment and ADG in heifer calves depending on birth weight suggests a dose-response effect rather than a true sex-specific effect of ZM on ADG. Hence, low birth weight calves (including heifers) may require a lower dose of ZM to mitigate any negative effect of what is otherwise a suitable dose for higher birth weight calves. These findings are in agreement with a previous randomized clinical trial testing the effect of daily oral zinc in diarrheic neonatal Holstein calves which, showed that ZM-treated calves had a numerically, though not significantly increased ADG compared to calves treated with zinc oxide or placebo due to small sample size [11]. In general, our trial findings are in agreement with the large body of human literature supporting the use of oral zinc for the prevention and treatment of diarrhea and impaired growth in children [5, 10, 33].

Zinc supplementation is widely accepted by global health organizations as a vital component of therapy for childhood diarrhea [3, 4], however, recent reviews of the literature demonstrated heterogeneity in study results on the basis of age, baseline zinc status, geographic location, and supplementation regimen [10, 34]. Similar to our findings, a sex-specific response to zinc supplementation has been demonstrated in several human studies. Zinc gluconate administered for diarrhea prevention reduced the incidence of dysentery in treated boys but not girls [35]; when given therapeutically, it reduced diarrhea duration and frequency more dramatically in boys compared to girls [36]. Similarly, zinc sulfate was shown to improve diarrhea outcomes in boys but improved growth rates in girls [13]. Broadly, these differences between male and female responses to zinc supplementation are not understood, though theories regarding differences in immune function and response [13, 35], diarrhea etiology [13], and nutrient requirements [35] have been proposed. In the current study, ZM-treated bulls demonstrated increased ADG compared to placebo-treated bulls while ZM-treated heifers demonstrated decreased ADG compared to placebo-treated heifers. However, due to a significant interaction between ZM treatment and birth weight, this reduction in ADG in ZM-treated heifers was overcome with increasing birth weight, such that ZM-treated heifers with birth weights above 42 kg experienced increased ADG during the pre-weaning period, compared to placebo-treated heifers with birth weights above 42 kg.

Differences in the growth response to ZM supplementation between bull and heifer calves may have been related to its effect on feed intake. Previous research on the effects of feeding various doses of oral zinc oxide to pre-ruminant dairy calves demonstrated that high levels of oral zinc supplementation resulted in reduced feed intake [23]. In the current trial, oral ZM dose was estimated to be significantly higher in heifers compared to bulls due to the significantly lower birth weight of heifers. Additionally, serum zinc concentrations in ZM-treated heifers were numerically higher than that of bulls, though this difference was not significant, likely due to the small sample size. Perhaps the higher zinc dose in heifers was associated with reduced feed intake, leading to reduced growth, and that this effect was more pronounced for ZM compared to ZS. The fact that ZM-treated heifers with birth weights approaching those of average bull calves (and, therefore, a similar zinc dose to that in bulls) experienced an increase in ADG over placebo-treated heifers similar to that of bull calves partially supports this theory. Although management practices on the study dairy were designed to be identical for both bulls and heifers, it is possible that subtle, unrecognized differences in nutritional and health management may also have contributed to sex-specific differences in weight gain. Nevertheless, future trials are warranted to investigate the potential differences in the dose-response to zinc supplementation between bulls and heifers.

We hypothesized that ADG would be increased in zinc-supplemented calves compared to placebo-supplemented calves due to the potential preventive and therapeutic effects of zinc supplementation on neonatal diarrhea. In other words, calf diarrhea is mitigated by zinc supplementation and, therefore, on the causal pathway between zinc and ADG. However, considering the similarly-reduced hazard of diarrhea and increased hazard of cure from diarrhea in both ZM and ZS treatment groups but a lack of effect of ZS on ADG, it is likely that the effect of ZM on ADG is not solely mediated through its effects on diarrhea. Differences in effectiveness between organic and inorganic formulations also may exist. In fact, the underlying mechanism of action of oral zinc remains unknown [6]. Several theories of the mechanisms of action of zinc in the prevention and treatment of childhood diarrhea exist, including a mucosal-protective role, a diarrhea-induced zinc deficiency, an essential element in cell-mediated immunity, and a modifier of intra-luminal electrolyte secretion and absorption [6, 37–39].

The clinical and practical implications of effects of ZM supplementation on ADG and diarrhea must be considered. Pre-weaned calf diarrhea remains an ongoing issue for the dairy industry. The deleterious effects on calf health and performance and the resulting economic burden create a strong incentive to treat and prevent diarrhea in pre-weaned calves. On large dairy operations like those in California’s Central Valley, small changes in disease incidence and duration as well as animal growth and performance can have profound economic consequences. As a non-antimicrobial product, zinc may become increasingly attractive as antimicrobials in livestock feed are under increased scrutiny and regulation due to concerns about antimicrobial resistance [2, 40]. Prevalence of *C*. *parvum* fecal shedding in a random sample of 92 study calves at onset and resolution of diarrhea was significantly higher in calves treated with zinc compared to Placebo-treated calves. In contrast, a previous study where calves that tested positive for *C*. *parvum* at the start of diarrhea and were treated with ZM had 16 times higher odds of being fecal ELISA negative at exit compared to the Placebo group (*P* = 0.08; power = 72.3%) [11]. The difference in findings may be due to the differences in the timing of diarrhea across treatment groups. For the current study’s random sample of calves that acquired, survived, and were sampled on the correct days, the mean age of calves on both onset and resolution of diarrhea was higher for ZM and ZS calves compared to placebo-treated calves. Although *C*. *parvum* oocyst shedding in infected calves can occur as early as 3 days of age, peak shedding occurs at about 14 days of age [41]. It is possible that the increase in prevalence of *C*. *parvum* shedding in ZM and ZS treated groups was due to the increased age of zinc-treated calves compared to placebo-treated calves at resolution of diarrhea. The latter explanation is also supported by our findings that the odds of microbiological cure from *C*. *parvum* significantly decreased in older calves, with no significant differences in the odds of cure between treatment groups. In addition, the current testing did not estimate the concentration of *C*. *parvum* shedding which may still differ between treatment groups.

Despite the large sample size, the current trial was limited to a single California dairy, which may represent other large dairies but does not reflect all the dairy management systems in California or elsewhere. Additionally, our results show that calves respond to zinc supplementation for diarrhea prevention differently depending on chemical formulation and calf sex. The latter could be due to differences in body weight between bulls and heifers and may point towards the need for sex-specific dosing. Furthermore, the current research did not evaluate the potential economic utility of zinc supplementation. Future studies on more accurate dosing of zinc by calf sex, the practical feasibility of weight-based dosing, and the expected cost-effectiveness of zinc administration as part of the management of pre-weaned dairy calves are warranted. Finally, our clinical trial was performed on a single, large, predominately Holstein, California dairy over a six-month period, which precluded our ability to evaluate differences due to season or breed. Hence, future studies to assess any modifying effect of breed and seasonal differences on the effect of zinc on calf health and weight gain are also needed.

## Conclusions

The current double blind, block-randomized placebo controlled clinical trial tested the effect of a prophylactic daily oral zinc supplementation in neonatal Holstein calves. Bull calves treated with ZM had a significantly increased ADG (22 g per day) during the pre-weaning period compared to placebo-treated bulls. In comparison, ZM-treated heifers had significantly lower average daily gain (9 g per day) compared to placebo-treated heifers, although higher ZM doses in low birthweight heifers may explain the lower ADG. Calves treated with either ZM or ZS had significantly lower risks of diarrhea and significantly higher risk of cure from diarrhea over the first 30 days of life compared to placebo-treated calves and hence the current trial demonstrated that zinc supplementation delayed diarrhea and expedited diarrhea recovery in pre-weaned calves. Additionally, zinc improved weight gain differentially in bulls compared to heifers, indicating the need for further research to investigate zinc dosing in calves.

## Supporting information

S1 Table Dietary milk components for pre-weaned calves from a double-blind block-randomized clinical trial. (DOCX)

S2 Table Ingredients of oral electrolyte solutions fed to pre-weaned calves from a double-blind block-randomized clinical trial. (DOCX)

S3 Table Estimate of total daily dietary zinc consumption of pre-weaned dairy calves during the first 14 days of life based on estimated daily consumption and laboratory-measured zinc content of dietary components. (DOCX)

S4 Table Characteristics and most likely causes of death based on field necropsy of pre-weaned dairy calves that died during a double-blind block-randomized clinical trial. (DOCX)

S5 Table Mean zinc concentrations (ppm) from milk samples treated with placebo, zinc methionine, or zinc sulfate, collected daily throughout the study period, and pooled by week and by treatment group from a double-blind block-randomized clinical trial. (DOCX)

S6 Table Comparison of oral zinc dose at start and end of treatment between neonatal Holstein bull and heifer calves treated with zinc methionine and zinc sulfate using ANOVA from a double-blind block-randomized clinical trial. (DOCX)

S7 Table Mean serum zinc concentrations in a random sample of neonatal Holstein bull and heifer calves (n = 36) pre- and post-treatment with placebo, zinc methionine, or zinc sulfate from a double-blind block-randomized clinical trial (N = 1,482). (DOCX)

S8 Table Comparison of fecal pathogen prevalence on the first day of diarrhea for randomly-sampled calves (N = 92) by treatment group using a Fisher Exact test from a double-blind block-randomized clinical trial. (DOCX)

S9 Table Comparison of fecal pathogen prevalence at resolution of clinical diarrhea for randomly-sampled calves (n = 92) by treatment group using a Fisher Exact test from a double-blind block-randomized clinical trial. (DOCX)

S10 Table Final linear regression model from a double-blind block-randomized clinical trial on the effect of treatment with zinc methionine (ZM) or zinc sulfate (ZS) compared to placebo on average daily gain in kilograms in neonatal Holstein heifer calves (n = 808) during the pre-weaning period. (DOCX)

S11 Table Final linear regression model from a double-blind block-randomized clinical trial on the effect of treatment with zinc methionine (ZM) or zinc sulfate (ZS) compared to placebo on average daily gain in kilograms in neonatal Holstein bull calves (n = 613) during the pre-weaning period. (DOCX)

S1 Dataset Raw data collected from trial, organized as separate excel sheets for enrollment, daily assessment, serum total protein, birth weight, exit treatment weight, exit trial weight, serum zinc testing, fecal samples, fecal testing, milk testing, and dead calves. (XLSX)
