# Supplementary material for: Effectiveness of zinc supplementation on diarrhea and average daily gain in pre-weaned dairy calves: A double-blind, block-randomized, placebo-controlled clinical trial
Source: PLoS One. 2019 Jul 10;14(7):e0219321. doi: 10.1371/journal.pone.0219321 (PMC6619766; doi:10.1371/journal.pone.0219321)
Supplement: S1 Table — (DOCX) [file pone.0219321.s001.docx]

**S1 Table**. **Dietary milk components for pre-weaned calves from a double-blind block-randomized clinical trial.**

| Calf Age (days) | Component | Product Use | Product Information |
| --- | --- | --- | --- |
| 0 – 25 | Pasteurized waste milk | Milk base | Dairy- and day-specific |
| 0 – 25 | Milk replacer powder | Milk base | 28% protein, 25% fat, non-medicated  (Strauss Feeds LLC, Watertown, WI) |
| 0 – 25 | Pennchlor 64 | Milk supplement | Chlortetracycline hydrochloride (64 g/lb)  (Pharmgate Animal Health, Omaha, NE) |
| 0 – 25 | NeoMed 325 | Milk supplement | Neomycin sulfate (325 g/lb)  (Bimeda, Inc., Le Sueur, MN) |
| 0 – 25 | Red Cell | Iron/Vitamin/Mineral supplement | (Farnam Companies, Inc., Phoenix, AZ) |
| 25 – weaning | Pasteurized waste milk | Milk base | -- |
| 25 – weaning | Milk replacer powder | Milk base | 28% protein, 25% fat, non-medicated  (Strauss Feeds LLC, Watertown, WI) |
| 25 – weaning | NT-10G | Medicated premix | Neomycin sulfate (10 g/lb)  Oxytetracycline hydrochloride (10 g/lb)  (Agri-Best^TM^, manufactured for Strauss Feeds LLC, Watertown, WI) |
| 25 – weaning | Prostart Plus | Probiotic milk supplement | (Agri-Best^TM^, manufactured for Strauss Feeds LLC, Watertown, WI) |
| 25 – weaning | Clarifly | Larvacidal milk supplement | (Central Life Sciences Inc, Dallas, TX) |
