# Supplementary material for: Effectiveness of zinc supplementation on diarrhea and average daily gain in pre-weaned dairy calves: A double-blind, block-randomized, placebo-controlled clinical trial
Source: PLoS One. 2019 Jul 10;14(7):e0219321. doi: 10.1371/journal.pone.0219321 (PMC6619766; doi:10.1371/journal.pone.0219321)
Supplement: S2 Table — (DOCX) [file pone.0219321.s002.docx]

**S2 Table**. **Ingredients of oral electrolyte solutions fed to pre-weaned calves from a double-blind block-randomized clinical trial.**

| Product | Ingredients |
| --- | --- |
| Calva™ electrolytes | Dextrose, Sodium Chloride, Sodium Bicarbonate, Potassium Chloride, Citric Acid, Glycine, Dried Fermentation Products of: Bacillus Subtilus, Lactobacillus acidophillus, Enterococcus faecium, Bifidobacterium bifidum; Ascorbic Acid, Vitamin E Supplement, Thiamine Mononitrate, Riboflavin Supplement, Pyridoxine Hydrochloride, Vitamin B12 Supplement, Biotin, Niacinamide, Calcium Pantothenate, Menadione Sodium Bisulfite Complex, Monosodium Phosphate, Magnesium Sulfate, Manganese Sulfate, Zinc Sulfate, Copper Sulfate, Vitamin A Supplement, Vitamin D3 Supplement, Mannanoligosaccharide, Artificial and Natural Color and Flavoring. |
| NuLife™ electrolytes | Glucose, Corn Starch, Sodium Acetate, Potassium Chloride, Glycine, Silicon Dioxide, Magnesium Stearate, and Artificial Coconut Flavor. |
