# Supplementary material for: Effectiveness of zinc supplementation on diarrhea and average daily gain in pre-weaned dairy calves: A double-blind, block-randomized, placebo-controlled clinical trial
Source: PLoS One. 2019 Jul 10;14(7):e0219321. doi: 10.1371/journal.pone.0219321 (PMC6619766; doi:10.1371/journal.pone.0219321)
Supplement: S3 Table — (DOCX) [file pone.0219321.s003.docx]

**S3 Table**. **Estimate of total daily dietary zinc consumption of pre-weaned dairy calves during the first 14 days of life based on estimated daily consumption and laboratory-measured zinc content of dietary components.**

| Dietary Component | Daily Consumption | Zinc Content  (mg/kg) | Maximum Daily Zinc Consumed  (mg) |
| --- | --- | --- | --- |
| Liquid milk | 4 L | 6.4 | 25.6 |
| Water | Minimal | 0.03 | < 0.10 |
| Calf starter grain | 0.15 kg^1^  (dry matter basis) | 140 (stainless bucket)  160 (galvanized bucket) | 24 |
| NuLife™ electrolytes | 2 L  (maximum) | 0 | 0 |
| Calva™ electrolytes | 2 L  (maximum) | 0 | 0 |

^1^Estimated intake of starter grain by calves less than 14 days of age (Bach et al., 2007; Khan et al., 2007).
