# Supplementary material for: Effectiveness of zinc supplementation on diarrhea and average daily gain in pre-weaned dairy calves: A double-blind, block-randomized, placebo-controlled clinical trial
Source: PLoS One. 2019 Jul 10;14(7):e0219321. doi: 10.1371/journal.pone.0219321 (PMC6619766; doi:10.1371/journal.pone.0219321)
Supplement: S4 Table — (DOCX) [file pone.0219321.s004.docx]

**S4 Table**. **Characteristics and most likely causes of death based on field necropsy of pre-weaned dairy calves that died during a double-blind block-randomized clinical trial.**

| Index ID | Sex | Treatment^1^ | Age at Death (d) | Presence of VSD^2^ | Cause of Death Based on Gross Field Necropsy |
| --- | --- | --- | --- | --- | --- |
| 1 | Bull | ZM | 50 | No | Enteritis |
| 2 | Bull | ZS | 65 | No | Proximal small intestinal obstruction |
| 3 | Bull | ZM | 54 | No | Abomasal torsion |
| 4 | Heifer | ZS | 17 | No | Euthanasia, pneumonia, sepsis |
| 5 | Heifer | Placebo | 86 | No | Euthanasia, chronic wasting, starvation |
| 6 | Bull | ZM | 38 | No | Pneumonia |
| 7 | Heifer | ZM | 14 | Yes | Omphalitits, septic peritonitis, sepsis |
| 8 | Bull | Placebo | 26 | No | Omphalitits, septic peritonitis, septic arthritis |
| 9 | Bull | Placebo | 31 | Yes | Sepsis |
| 10 | Bull | ZM | 63 | Yes | Congestive heart failure secondary to VSD |
| 11^3^ | Bull | ZM | 55 | -- | -- |
| 12 | Heifer | ZM | 18 | Yes | Congestive heart failure secondary to VSD |
| 13 | Bull | Placebo | 17 | Yes | Congestive heart failure secondary to VSD |
| 14 | Bull | ZS | 54 | No | Abomasal torsion |
| 15 | Bull | ZM | 43 | Yes | Congestive heart failure secondary to VSD |
| 16 | Bull | Placebo | 22 | Yes | Omphalitits, septic peritonitis |
| 17 | Heifer | ZM | 73 | No | Hemorrhagic gastroenteritis |
| 18 | Bull | ZM | 51 | No | Pneumonia |
| 19 | Heifer | ZM | 16 | Yes | Euthanasia, fibrinous pleuropneumonia |
| 20 | Bull | ZS | 33 | Yes | Congestive heart failure secondary to VSD |
| 21 | Heifer | ZS | 51 | No | Mesenteric torsion, abomasal rupture, toxic peritonitis |

^1^Treatments: placebo = 0.44 g fresh milk replacer powder (MRP); zinc methionine = 80 mg of zinc (0.45 g zinc methionine complex as Zinpro180) in 0.44 g of fresh MRP; zinc sulfate = 80 mg of zinc (0.22 g zinc sulfate monohydrate) in 0.44 g of fresh MRP.

^2^VSD = ventral septal defect.

^3^Calf was picked up by rendering truck before field necropsy could be performed.
