# Supplementary material for: Effectiveness of zinc supplementation on diarrhea and average daily gain in pre-weaned dairy calves: A double-blind, block-randomized, placebo-controlled clinical trial
Source: PLoS One. 2019 Jul 10;14(7):e0219321. doi: 10.1371/journal.pone.0219321 (PMC6619766; doi:10.1371/journal.pone.0219321)
Supplement: S5 Table — (DOCX) [file pone.0219321.s005.docx]

**S5 Table**. **Mean zinc concentrations (ppm) from milk samples treated with placebo, zinc methionine, or zinc sulfate, collected daily throughout the study period, and pooled by week and by treatment group from a double-blind block-randomized clinical trial.**

| Treatment^1^ | Pools, n | Mean | SE | 95% CI |  |
| --- | --- | --- | --- | --- | --- |
|  |  |  |  | Lower | Upper |
| Placebo | 16 | 6.33^b^ | 0.218 | 5.90 | 6.75 |
| Zinc methionine | 16 | 45.94^a^ | 0.755 | 44.46 | 47.42 |
| Zinc sulfate | 16 | 45.81^a^ | 0.660 | 44.52 | 47.11 |

^a-b^Means with different superscripts within columns are significantly different (P < 0.05) according to the Kruskal-Wallis Rank Sum test and Post-hoc Nemenyi-tests.

^1^Treatments: placebo = 0.44 g fresh milk replacer powder (MRP); zinc methionine = 80 mg of zinc (0.45 g zinc methionine complex as Zinpro180) in 0.44 g of fresh MRP; zinc sulfate = 80 mg of zinc (0.22 g zinc sulfate monohydrate) in 0.44 g of fresh MRP.
