# Supplementary material for: Effectiveness of zinc supplementation on diarrhea and average daily gain in pre-weaned dairy calves: A double-blind, block-randomized, placebo-controlled clinical trial
Source: PLoS One. 2019 Jul 10;14(7):e0219321. doi: 10.1371/journal.pone.0219321 (PMC6619766; doi:10.1371/journal.pone.0219321)
Supplement: S6 Table — (DOCX) [file pone.0219321.s006.docx]

**S6 Table**. **Comparison of oral zinc dose at start and end of treatment between neonatal Holstein bull and heifer calves treated with zinc methionine and zinc sulfate using ANOVA from a double-blind block-randomized clinical trial.**

| Zinc methionine^1^ | Bulls | | | | | Heifers | | | | |
| --- | --- | --- | --- | --- | --- | --- | --- | --- | --- | --- |
|  | n | Mean | SE | 95% CI | | n | Mean | SE | 95% CI | |
|  |  |  |  | Lower | Upper |  |  |  | Lower | Upper |
| Start zinc dose (mg/kg) | 202 | 1.88^b^ | 0.018 | 1.85 | 1.92 | 289 | 2.11^a^ | 0.018 | 2.07 | 2.14 |
| End zinc dose (mg/kg) | 202 | 1.76^b^ | 0.014 | 1.73 | 1.79 | 287 | 1.94^a^ | 0.013 | 1.92 | 1.97 |
| Zinc sulfate^2^ | Bulls | | | | | Heifers | | | | |
|  | n | Mean | SE | 95% CI | | n | Mean | SE | 95% CI | |
|  |  |  |  | Lower | Upper |  |  |  | Lower | Upper |
| Start zinc dose (mg/kg) | 221 | 1.85^b^ | 0.016 | 1.82 | 1.88 | 270 | 2.10^a^ | 0.018 | 2.06 | 2.13 |
| End zinc dose (mg/kg) | 221 | 1.73^b^ | 0.013 | 1.71 | 1.76 | 269 | 1.95^a^ | 0.014 | 1.92 | 1.97 |

^a-b^Means with different superscripts within rows are significantly different (P < 0.05) according to ANOVA.

^1^Zinc methionine = 80 mg of zinc (0.45 g zinc methionine complex as Zinpro180) in 0.44 g of fresh MRP.

^2^Zinc sulfate = 80 mg of zinc (0.22 g zinc sulfate monohydrate) in 0.44 g of fresh MRP.
