# Supplementary material for: Effectiveness of zinc supplementation on diarrhea and average daily gain in pre-weaned dairy calves: A double-blind, block-randomized, placebo-controlled clinical trial
Source: PLoS One. 2019 Jul 10;14(7):e0219321. doi: 10.1371/journal.pone.0219321 (PMC6619766; doi:10.1371/journal.pone.0219321)
Supplement: S7 Table — (DOCX) [file pone.0219321.s007.docx]

**S7 Table**. **Mean serum zinc concentrations in a random sample of neonatal Holstein bull and heifer calves (n=36) pre- and post-treatment with placebo, zinc methionine, or zinc sulfate from a double-blind block-randomized clinical trial (n=1,482).**

| Placebo^1^ | Bulls | | | | | Heifers | | | | |
| --- | --- | --- | --- | --- | --- | --- | --- | --- | --- | --- |
|  | n | Mean | SE | 95% CI | | n | Mean | SE | 95% CI | |
|  |  |  |  | Lower | Upper |  |  |  | Lower | Upper |
| Pre-treatment serum zinc (ppm) | 8 | 1.00^a^ | 0.133 | 0.74 | 1.26 | 4 | 0.93^a^ | 0.078 | 0.77 | 1.08 |
| Post-treatment serum zinc (ppm) | 8 | 1.11^a^ | 0.114 | 0.88 | 1.33 | 4 | 0.98^a^ | 0.082 | 0.82 | 1.14 |
| Zinc methionine^2^ | Bulls | | | | | Heifers | | | | |
|  | n | Mean | SE | 95% CI | | n | Mean | SE | 95% CI | |
|  |  |  |  | Lower | Upper |  |  |  | Lower | Upper |
| Pre-treatment serum zinc (ppm) | 6 | 1.21^a^ | 0.117 | 0.98 | 1.44 | 6 | 1.14^a^ | 0.213 | 0.72 | 1.56 |
| Post-treatment serum zinc (ppm) | 6 | 1.57^b^ | 0.187 | 1.38 | 1.75 | 6 | 1.87^b^ | 0.196 | 1.48 | 2.25 |
| Zinc sulfate^3^ | Bulls | | | | | Heifers | | | | |
|  | n | Mean | SE | 95% CI | | n | Mean | SE | 95% CI | |
|  |  |  |  | Lower | Upper |  |  |  | Lower | Upper |
| Pre-treatment serum zinc (ppm) | 3 | 0.99^a^ | 0.413 | 0.18 | 1.80 | 9 | 0.90^a^ | 0.107 | 0.69 | 1.11 |
| Post-treatment serum zinc (ppm) | 3 | 1.63^ab^ | 0.145 | 1.35 | 1.92 | 9 | 1.52^b^ | 0.172 | 1.35 | 1.69 |

^a-f^Means with different superscripts within rows and columns are significantly different (P < 0.05) according to ANOVA.

^1^Placebo = 0.44 g fresh milk replacer powder (MRP).

^2^Zinc methionine = 80 mg of zinc (0.45 g zinc methionine complex as Zinpro180) in 0.44 g of fresh MRP.

^3^Zinc sulfate = 80 mg of zinc (0.22 g zinc sulfate monohydrate) in 0.44 g of fresh MRP.
