# Supplementary material for: Effectiveness of zinc supplementation on diarrhea and average daily gain in pre-weaned dairy calves: A double-blind, block-randomized, placebo-controlled clinical trial
Source: PLoS One. 2019 Jul 10;14(7):e0219321. doi: 10.1371/journal.pone.0219321 (PMC6619766; doi:10.1371/journal.pone.0219321)
Supplement: S8 Table — (DOCX) [file pone.0219321.s008.docx]

**S8 Table**. **Comparison of fecal pathogen prevalence on the first day of diarrhea for randomly-sampled calves (n=92) by treatment group using a Fisher Exact test from a double-blind block-randomized clinical trial.**

| Fecal Pathogen^1^ | Treatment^2^ | | | | | | | | | | | | |
| --- | --- | --- | --- | --- | --- | --- | --- | --- | --- | --- | --- | --- | --- |
|  | Placebo (n=38) | | | | Zinc methionine (n=30) | | | | Zinc sulfate (n=24) | | | |  |
|  | n (%) | SE | 95% CI | | n (%) | SE | 95% CI | | n (%) | SE | 95% CI | | *P* value |
|  |  |  | Lower | Upper |  |  | Lower | Upper |  |  | Lower | Upper |  |
| K99^3,4^ | 2 (22.2%) | 0.139 | -0.05 | 0.49 | 3 (30.0%) | 0.145 | 0.02 | 0.58 | 4 (44.4%) | 0.166 | 0.12 | 0.77 | 0.694 |
| Rota^5^ | 26 (68.4%) | 0.075 | 0.54 | 0.83 | 16 (53.3%) | 0.091 | 0.35 | 0.71 | 17 (70.8%) | 0.093 | 0.53 | 0.89 | 0.331 |
| Corona^6^ | 1 (2.6%) | 0.026 | -0.02 | 0.08 | 2 (6.7%) | 0.046 | -0.02 | 0.16 | 1 (4.2%) | 0.041 | -0.04 | 0.12 | 0.819 |
| Crypto^7^ | 15 (39.5%) | 0.079 | 0.24 | 0.55 | 13 (43.3%) | 0.090 | 0.26 | 0.61 | 12 (50.0%) | 0.102 | 0.30 | 0.70 | 0.719 |

^1^Fecal pathogen detection was performed using a commercial kit (Pathasure Enteritis 4; Biovet, Quebec, Canada).

^2^Treatments: placebo = 0.44 g fresh milk replacer powder (MRP); zinc methionine = 80 mg of zinc (0.45 g zinc methionine complex as Zinpro180) in 0.44 g of fresh MRP; zinc sulfate = 80 mg of zinc (0.22 g zinc sulfate monohydrate) in 0.44 g of fresh MRP.

^3^*E. coli* K99.

^4^The number of calves in each treatment group tested for E. coli K99: placebo (n=9), zinc methionine (n=10), zinc sulfate (n=9).

^5^Rotavirus.

^6^Coronvirus.

^7^*Cryptosporidium parvum*.
