# Supplementary material for: Effectiveness of zinc supplementation on diarrhea and average daily gain in pre-weaned dairy calves: A double-blind, block-randomized, placebo-controlled clinical trial
Source: PLoS One. 2019 Jul 10;14(7):e0219321. doi: 10.1371/journal.pone.0219321 (PMC6619766; doi:10.1371/journal.pone.0219321)
Supplement: S9 Table — (DOCX) [file pone.0219321.s009.docx]

**S9 Table**. **Comparison of fecal pathogen prevalence at resolution of clinical diarrhea for randomly-sampled calves (n=92) by treatment group using a Fisher Exact test from a double-blind block-randomized clinical trial.**

| Fecal Pathogen^1^ | Treatment^2^ | | | | | | | | | | | | |
| --- | --- | --- | --- | --- | --- | --- | --- | --- | --- | --- | --- | --- | --- |
|  | Placebo (n=38) | | | | Zinc methionine (n=30) | | | | Zinc sulfate (n=24) | | | | |
|  | n (%) | SE | 95% CI | | n (%) | SE | 95% CI | | n (%) | SE | 95% CI | | *P* value |
|  |  |  | Lower | Upper |  |  | Lower | Upper |  |  | Lower | Upper |  |
| K99^3,4^ | 2 (22.2%) | 0.139 | -0.05 | 0.49 | 6 (60.0%) | 0.155 | 0.30 | 0.90 | 5 (55.6%) | 0.166 | 0.23 | 0.88 | 0.256 |
| Rota^5^ | 18 (47.4%) | 0.081 | 0.31 | 0.63 | 9 (30.0%) | 0.084 | 0.14 | 0.46 | 9 (37.5%) | 0.099 | 0.18 | 0.57 | 0.344 |
| Corona^6^ | 1 (2.6%) | 0.026 | -0.02 | 0.08 | 1 (3.3%) | 0.033 | -0.03 | 0.10 | 0 (0.0%) | 0.000 | 0.00 | 0.00 | 1.000 |
| Crypto^7^ | 16 (42.1%) | 0.080 | 0.26 | 0.58 | 23 (76.7%) | 0.077 | 0.62 | 0.92 | 18 (75.0%) | 0.088 | 0.58 | 0.92 | 0.006 |

^1^Fecal pathogen detection was performed using a commercial kit (Pathasure Enteritis 4; Biovet, Quebec, Canada).

^2^Treatments: placebo = 0.44 g fresh milk replacer powder (MRP); zinc methionine = 80 mg of zinc (0.45 g zinc methionine complex as Zinpro180) in 0.44 g of fresh MRP; zinc sulfate = 80 mg of zinc (0.22 g zinc sulfate monohydrate) in 0.44 g of fresh MRP.

^3^*E. coli* K99.

^4^The number of calves in each treatment group tested for E. coli K99: placebo (n=9), zinc methionine (n=10), zinc sulfate (n=9).

^5^Rotavirus.

^6^Coronvirus.

*^7^Cryptosporidium parvum*.
