# Supplementary material for: Effectiveness of zinc supplementation on diarrhea and average daily gain in pre-weaned dairy calves: A double-blind, block-randomized, placebo-controlled clinical trial
Source: PLoS One. 2019 Jul 10;14(7):e0219321. doi: 10.1371/journal.pone.0219321 (PMC6619766; doi:10.1371/journal.pone.0219321)
Supplement: S10 Table — (DOCX) [file pone.0219321.s010.docx]

**S10 Table**. **Final linear regression model from a double-blind block-randomized clinical trial on the effect of treatment with zinc methionine (ZM) or zinc sulfate (ZS) compared to placebo on average daily gain in kilograms in neonatal Holstein heifer calves (n=808) during the pre-weaning period.**

| Variable | β | SE | 95% CI |  | *P*-value |
| --- | --- | --- | --- | --- | --- |
|  |  |  | Lower | Upper |  |
| Treatment^1^ |  |  |  |  |  |
| Placebo | Reference |  |  |  |  |
| Zinc methionine | -0.165 | 0.074 | -0.309 | -0.020 | 0.026 |
| Zinc sulfate | -0.108 | 0.072 | -0.248 | 0.033 | 0.134 |
| Milk volume^2^ | 0.002 | 0.001 | 0.001 | 0.004 | 0.009 |
| Birth weight (kg) | -0.002 | 0.001 | -0.004 | 0.001 | 0.123 |
| ZM x Birth weight | 0.004 | 0.001 | 0.000 | 0.008 | 0.037 |
| ZS x Birth weight | 0.002 | 0.002 | -0.001 | 0.006 | 0.173 |
| Intercept | 0.444 | 0.133 | 0.183 | 0.706 | 0.001 |

^1^Treatments: placebo = 0.44 g fresh milk replacer powder (MRP); zinc methionine = 80 mg of zinc (0.45 g zinc methionine complex as Zinpro180) in 0.44 g of fresh MRP; zinc sulfate = 80 mg of zinc (0.22 g zinc sulfate monohydrate) in 0.44 g of fresh MRP.

^2^Total milk volume (L) fed to calves during the assessment period.
